# Observations on Bleeding

**Published:** 1826-07

**Authors:** 


### ON BLEEDING.

Referring to the observations in the last Number of the Medico-Chirurgical Review, on the Pulse, (Review of Dr. Butter's Work) as indicating the propriety of bleeding, the following memoranda may perhaps be considered as not irrelevant.

In an affection of the head, with great tightness across the forehead—where the pulse was of a wiry hardness, contracted, and in frequency 120, bleeding generally and locally, purgatives and sudorifics, had been used for some days before the writer saw the patient;—the above state of pulse continuing, seemed to indicate a continuance of the plan; bleeding was twice repeated—pediluvia used, and the nape of the neck blistered, without any diminution of symptoms. The long-continued want of rest induced the writer to direct a dose of æther, compound camphorated tincture, and camphor mixture, to be given at night. The effect was favourable—soothing the patient and producing so favourable a change in the pulse, as to induce a repetition of moderate doses of the same remedies every four hours, which terminated in complete recovery.

Sometime afterwards, the writer was called to a patient far advanced in pregnancy, in whom nearly similar symptoms had been under treatment for several days—by local and general depletion—daily purgatives—sudorifics, without removal of the pain, or reduction either of the frequency or hardness of the pulse; the former case was mentioned to the two gentlemen in attendance, (who contemplated a fatal issue of the case,) and the same means were resorted to with similar success. Yet in neither case would such means have been justified, or would the omission of bleeding have been warranted in the first instance.

The writer has witnessed in an extensive degree the treatment of the epidemic cholera in India, and can safely affirm, that death has in many instances been hastened by bleeding; which is not surprising, for never were means used more empirically than in this disease, and *even by authority*—yet bleeding, where eighteen or twenty ounces could be obtained from an European, and ten to fourteen from a native, was *often* useful, (no treatment was uniformly useful) not only by restoring the balance of the circulation, but by taking off the irritability of stomach.

In the first instance in which the writer felt justified in using the lancet, the irritation of stomach ceased when about ten ounces of blood had been drawn, in a native woman, and the administration of internal remedies was attended with success.

Dr. Gregory (following the anti-mercurialists) has censured the use of blood-letting in this disease, unguardedly, if not unjustifiably—because he has not data on which to qualify, even a Professor, for censuring those who are eye-witnesses of *effect*. Thus medical men, who have not seen disease in Tropical climates, censure the use of the only remedy, by which these diseases can be cut short, and which it would be justifiable to use, were the bad effects, such as they are represented to be, but which they are not, when mercury is properly used.

Bleeding and mercury require judgment to decide, when, and to what extent they are to be used, but are not to be laid aside because theories are formed in opposition to the results of practical experience; or because they may be misused, amongst the numerous persons let loose on the world as practitioners.

P.S.—It may be worthy of notice as a fact, that in eighteen years residence in India, the writer only saw one case of stone in the bladder in a native—none in Europeans. MEDICUS.

